# The 40th Anniversary of IVF: Has ART’s Success Reached Its Peak?

**Published:** 2018

**Authors:** Mohammad Reza Sadeghi

This year, 25th July will be the 40th anniversary for birth celebration of Louise Joy Brown, the first IVF baby; Consequently, IVF will be forty years old. Therefore, simple technology has been the architect of wonderful development and subsequently originator of stunning technologies in the field of biomedicine such as stem cell technology, regenerative medicine, cloning and transgenic technologies, preimplantation genetics screening/diagnosis and more recently gene-editing technology to knock out pathogenic gene mutations in human embryos. In addition, the prospect of this area is also very promising and can be the basis of amazing changes in the treatment of severe illnesses such as cancer, obesity, diabetes, cardiovascular and neurodegenarative diseases. Along with all these merits and advancements, it must be acknowledged that infertility treatment still faces numerous challenges and problems most notably the low success rate and the consequent repeated implantation failure.

In essence, many factors can contribute to this problem, most of which are due to the nature of human reproduction and fertilization process, so the maximum chance of pregnancy per cycle is less than 20% in healthy couples. Another reason for this low success rate is related to *in vitro* performing of fertilization and embryo culture and the inability to accurately and consistently control many vital parameters for human gametes and embryo in accordance with the same *in vivo* condition. Are we really unable to accurately control these parameters? The fact is that technological advancement and the introduction of new equipment have facilitated optimal achievement in *in vitro* condition. In addition, quality control and continuous monitoring of the performance of equipment and staff and the quality of chemicals, reagents and plastic ware also help to achieve this propose. The subsequent question is whether IVF clinics have been successful in fulfilling these requirements and achieving maximum success rate.

According to annual data published by European Society of Human Reproduction and Embryology (ESHRE) in Europe and the Centers for Disease Control and Prevention (CDC) in the US from large ART fertility treatment registries, success rate of IVF clinics shows wide variation. Few top clinics are reporting their success rate to be more than 40%, while the rate of few others is less than 10% and a large number of clinics are between these two ranges. Unfortunately, even in developed countries, the number of clinics with a low success rate is very high. These clinics waste a large part of the health budget from the public resources and pocket of patients (1).

According to obtained data from Australia and New Zealand Assisted Reproduction Database (ANZARD) on success rates of IVF clinics, the qualified clinics (top 25th percentile) are spending about 2 million dollars of health resources to take home 100 live babies; however, low quality clinics (bottom 25th percentile) devote more than 6 million dollars for the same 100 live babies. This means that they spend three times more than qualified centers and also the patients in the lowest performing clinic must undergo seven IVF cycles more than the patients referring to the top clinic to achieve success. The failed couples provide a business for these poor-performing clinics since the patients have to resume their treatment due to inefficiency ofclinics and their extreme desire to leverage maximum benefits from the patients. Furthermore, most of low-performing clinics have no program for achieving better results. In general, community and health care providers in public and private sections have no idea who would provide good or bad IVF services (2).

It is estimated more than 400,000 babies from 1.6 million ART cycles are born around the world every year. Due to the changes in lifestyle indifferent societies, especially postponding marriage, first pregnancy and childbearing after the fourth decade of life, the need for assisted reproductive techniques and subsequently the cost of infertility treatment are increasing worldwide. According to the report published by CDC, the total market cost of fertility services was up to $3.5 billion in the U.S. in 2012. Regarding the data published in1988, it increased more than fourfold during 24 years and it still continues to grow. Therefore, intensive and continuous monitoring of IVF clinics by governmental authorities and nongovernmental agencies is necessary to maintain and improve the quality of infertility services and patients’ rights (3).

An effective tool for monitoring IVF clinics is the development of ART services registries. Inspection on the results of IVF clinics is a requirement for ART services which is accepted worldwide. Moreover, availability, efficiency, safety, and qualification of ART services are necessary for community, infertile couples, physicians and other professional members, and health policy makers to make universal trust on IVF clinics and ART outcomes. At recent, data reporting and IVF registry are performed in most of developed countries, so an international organization entitled the International Committee Monitoring ART (ICMART) has been established for this purpose. There was voluntarily collected data of 2184 clinics from 52 countries including the type of clinics, cycles and protocols, pregnancy rates, take-home babies, twin birth rates and pregnancy complications in the last report in 2004. Unfortunaly, most African and Asian countries do not report their data and even there is no national data registry for ART services in these countries (4).

Another important factor for improving ART success rate is rigorous monitoring of IVF clinics and their procedures through quality control and quality assurance as total quality management (TQM). TQM is a prerequisite for successful management of an IVF clinic. Unfortunately, a large number of centers are neglecting the role of TQM and also there are no established performance indicators for ART laboratories. In most clinics running TQM, no peer-based external inspection was provisioned to supervise its excellence implementation. At recent, College of American Pathologists (CAP) in collaboration with the American Society for Reproductive Medicine (ASRM) designed an accreditation program regarding the needs of IVF laboratories. In Europian countries, ESHRE has developed a certification program for centers through a careful evaluation of defined standards and on site visit. Skilled inspectors will assess centres according to ESHRE standards to provide advice for continuous improvement. However, this certification program and systemic inspection is not performed in most Asian and African countries (5).

In conclusion, it seems that expansion of national and international IVF registry and changing it from voluntary status to a mandatory duty and also monitoring the accuracy of reported data will be effective in efficiency promotion and performance transparency of IVF clinics. In addition, continuous monitoring and inspection of IVF clinics by authorities and strict enforcement on TQM implementation will definitely play a critical role in improvement of performance and protecting patients’ rights. The future is bright, since rigorous efforts of scientists and scholars have always solved most of complications and improve ART outcomes.
